# Supplementary material for: Immunonano-Lipocarrier-Mediated Liver Sinusoidal Endothelial Cell-Specific RUNX1 Inhibition Impedes Immune Cell Infiltration and Hepatic Inflammation in Murine Model of NASH
Source: Int J Mol Sci. 2021 Aug 6;22(16):8489. doi: 10.3390/ijms22168489 (PMC8395158; doi:10.3390/ijms22168489)
Supplement: Supplementary file 1 [file ijms-22-08489-s001.zip › ijms-1203328-supplementary.pdf]

Supplementary Materials

Table S1: Flow Cytometry Antibody Panel

<u>**Antibody**</u>	<u>**Antibody source**</u>	<u>**Fluorochrome**</u>
Goat Anti-RUNX1	Santa Cruz	Unconjugated
Rabbit Anti-CD31	Thermofisher Scientific	Unconjugated
Rabbit Anti-αSMA	Elabsciences	Unconjugated
VEGFR2 mouse monoclonal IgG	Santa Cruz	Unconjugated
CD45	BD Biosciences	FITC
CD3	eBiosciences	PE
CD11b	eBiosciences	Alexa405
F4/80	eBiosciences	FITC
Mouse Antigoat	Santa Cruz	PE
Goat Anti Rabbit	Santa Cruz	FITC
Goat anti-Mouse	Santa Cruz	TR
ICAM1	Invitrogen, Thermofisher Scientific	PE
VCAM1	Invitrogen, Thermofisher Scientific	PE

Supplementary Figure

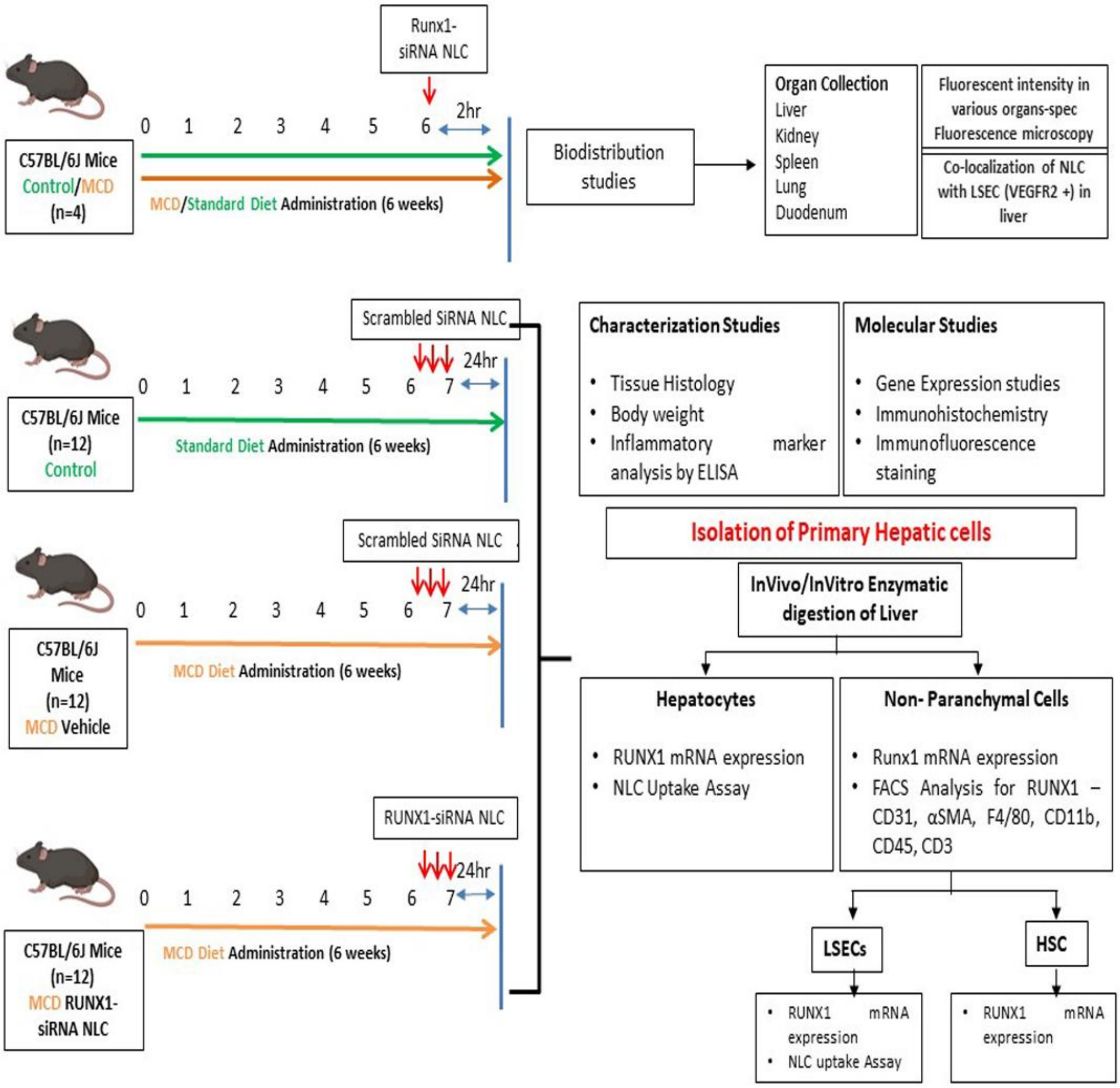

Figure S1: Schema of Animal Studies. hr: hours.

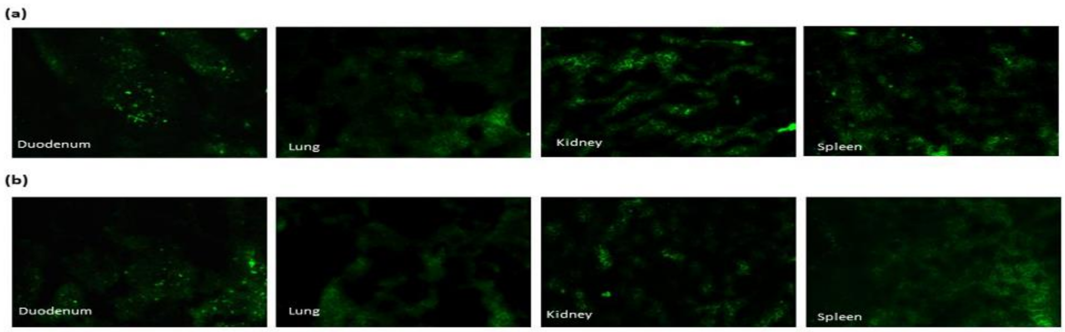

Figure S2: Florescence images (20x) showing in vivo biodistribution of RUNX1-siRNA NLC in different organs in (A) Control and (B) MCD mice.

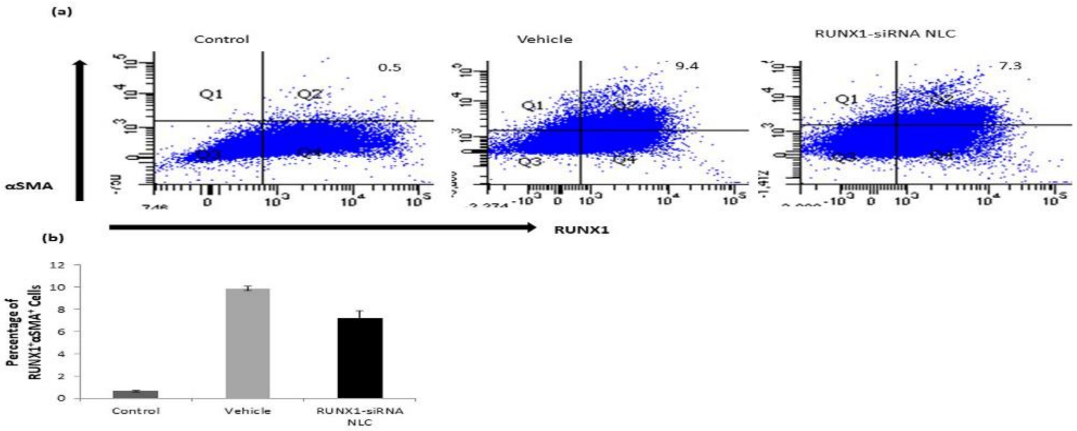

Figure S3: (A) Representative dot plots showing in RUNX1$^{+}\alpha$SMA^{+} cells. (B) Bar diagrams depicting average percentage of RUNX1$^{+}\alpha$SMA^{+} cells in vehicle and RUNX1-siRNA NLC mice. Data represent mean ± SD, n = 5.

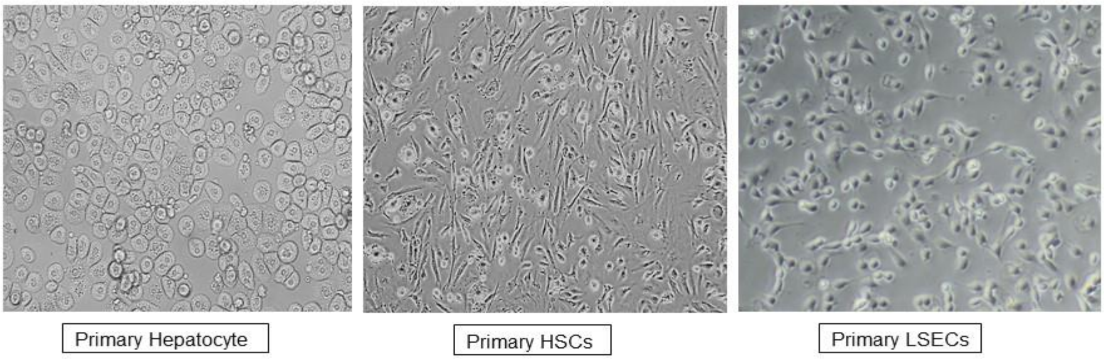

Figure S4: Cultured primary hepatic cells (Hepatocytes, HSCs, LSECs).

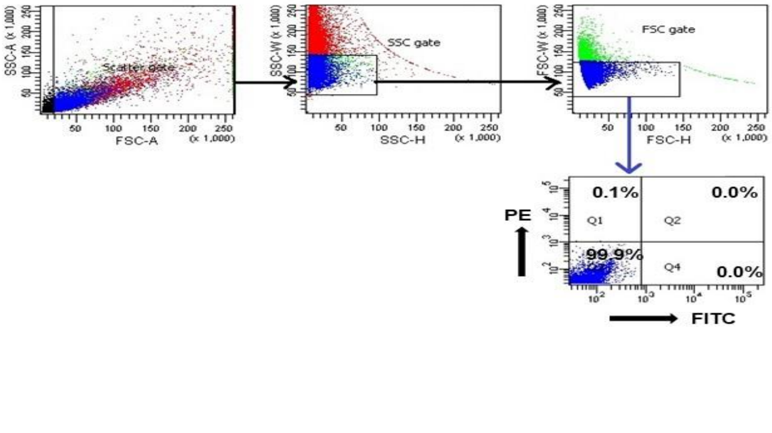

Figure S5: Gating strategies. Gating strategies employed to exclude cell doublets from flow cytometry data using forward scatter (FSC) and side scatter (SSC) gates. Events were passed through two successive gates of FSC area (FSC-A) by FSC width (FSC-W) and side scatter area (SSC-A) by SSC width (SSC-W) utilizing low pulse width

signal to select single cells. Further gates were set on dot plots for PE and FITC channels using unstained cells to subtract any background fluorescence. The gating strategies ensured the exclusion of cell doublets from flow cytometry data. First, events that deviated from the linear correlation between the forward scatter area (FSC-A) and the FSC height (FSC-H) parameters were excluded. Alternatively, events were passed through two successive gates of FSC-A by FSC width (FSC-W) and side scatter area (SSC-A) by SSC width (SSC-W) utilizing the low pulse width signal indicative of single cells. These methods were highly effective in eliminating the majority of contaminating doublets cells. The gates were set on the basis of the isotope control group. Further gates were set on dot plots for PE and FITC channels using unstained cells to subtract any background fluorescence.
